# Adjuvant immunochemotherapy with oral Tegafur/Uracil plus PSK in patients with stage II or III colorectal cancer: a randomised controlled study

**DOI:** 10.1038/sj.bjc.6601619

**Published:** 2004-03-02

**Authors:** S Ohwada, T Ikeya, T Yokomori, T Kusaba, T Roppongi, T Takahashi, S Nakamura, S Kakinuma, S Iwazaki, H Ishikawa, S Kawate, T Nakajima, Y Morishita

**Affiliations:** 1 Department of Surgery, Gunma University, Graduate School of Medicine, Gunma Oncology Study Group (GOSG), 3-39-15 Showa-Machi, Maebashi 371-8511, Gunma, Japan

**Keywords:** randomised controlled trial, colorectal cancer, PSK, tegafur/uracil, UFT

## Abstract

Intravenous fluorouracil and leucovorin is the standard adjuvant treatment for stage III colon cancer. However, oral adjuvant chemotherapy is attractive because it has low toxicity and greater convenience. We investigated the benefits of oral protein-bound polysaccharide K (PSK) with tegafur/uracil (UFT) as an adjuvant in stage II and III colorectal cancer. Patients were assigned to groups that received either 3 g PSK plus 300 mg UFT, or 300 mg UFT alone orally each day for a 2-year period following intravenous mitomycin C. Of 207 registered patients, 205 with stage II (*n*=123) or III (*n*=82) were analysed. The 5-year disease-free survival was 73.0% (95% CI 65.6–80.4%) with PSK (*n*=137) and 58.8% (95% CI 47.1–70.5%) in the controls (*n*=68) (*P*=0.016). Polysaccharide K reduced the recurrence by 43.6% (95% CI 4.5–66.7%) and mortality by 40.2% (95% CI −12.5 to 68.3%). The 5-year survival was 81.8% (95% CI 75.3–88.2%) in the PSK group and 72.1% (95% CI 61.4–82.7%) in the control group (*P*=0.056). In stage III patients, disease-free and overall survivals in patients receiving PSK were increased significantly: 60.0% (95% CI 47.1–72.9%) and 74.6% (95% CI 63.0–86.1%) in the PSK group as compared with 32.1% (95% CI 14.8–49.4%) and 46.4% (95% CI 28.0–64.9%) in the controls (*P*=0.002 and 0.003, respectively). Polysaccharide K prevented recurrence, particularly lung metastases (*P*=0.02; odds ratio 0.27; 95% CI 0.09–0.77). In the models, the presence of regional metastases (relative risk, 2.973; 95% CI 1.712–5.165; *P*<0.001), omission of PSK (relative risk, 2.106; 95% CI 1.221–3.633; *P*=0.007), and higher primary tumour (relative risk, 4.398; 95% CI 1.017–19.014; *P*=0.047) were each significant indicators of recurrence. Adverse effects were mild and compliance was good. Oral PSK with UFT reduced recurrence in stage II and III colorectal cancer, and increased survival in stage III.

Currently, the standard adjuvant treatment for stage III colon cancer is 5-fluorouracil plus leucovorin (5-FU/LV) for 6–8 months (Francini *et al*, 1994; IMPACT, 1995; O'Connell *et al*, 1998). In an attempt to improve the therapeutic index of 5-FU and its tolerability, research has focused on the study of oral 5-FU prodrugs. Tegafur/uracil (UFT®; Taiho Pharmaceutical Co., Japan) is an orally administered fluoropyrimidine inhibitor of dihydropyrimidine dehydrogenase, and contains tegafur and uracil in a 1 : 4 molar ratio. Tegafur is an orally bioavailable prodrug of 5-FU, and uracil reversibly inhibits the primary catabolic enzyme for 5-FU, namely dihydropyrimidine dehydrogenase. Phase I/II studies of advanced or metastatic colorectal cancer treated with different regimens of UFT and LV achieved excellent response rates and had favourable toxicity (Saltz *et al*, 1995; Feliu *et al*, 1997; Rustum, 1997). In phase III, oral UFT/LV provided a safer, more convenient oral alternative to a standard bolus i.v. regimen of 5-FU/LV for metastatic colorectal cancer, while producing equivalent survival (Carmichael *et al*, 2002; Douillard *et al*, 2002).

Protein-bound polysaccharide K (KRESTIN®; Kureha Chemical Industry Co., Japan), extracted from the mycelia of *Coriolus versicolor*, has immunomodulatory activities. It induces the production of interleukin (IL)-2 and interferon (*IFN*) *γ*, thereby stimulating lymphokine-activated killer cells (LAK) and enhancing natural killer (NK) cells (Hirose *et al*, 1990; Hayashida *et al*, 1991; Ebina and Murata, 1992; Kato *et al*, 1995; Yefenof *et al*, 1995; Algarra *et al*, 1997; Harada *et al*, 1997; Pedrinaci *et al*, 1999). We have reported that adjuvant therapy with PSK increases the levels of cytotoxic T cells, helper T cells, and NK cells in patients with resected colorectal cancer (Ohwada *et al*, 1994).

Protein-bound polysaccharide K and UFT are popularly used for adjuvant immunochemotherapy in gastric cancer patients in Japan. In a few reports, PSK and UFT have been used as adjuvant chemotherapy in colorectal cancer. A randomised, double-blind trial of stage III and IV colorectal cancers using the rules for colorectal cancer in Japan (this stage corresponds to AJCC stage III) showed that PSK provided significant benefits in improving disease-free and overall survival. The beneficial effects of PSK have been attributed to the activation of leucocyte chemotactic locomotion and phagocytic activity (Torisu *et al*, 1990). Adjuvant chemotherapy with PSK for over 3 years, and oral 5-FU for 2 years, following intravenous mitomycin C (MMC®), significantly increased the disease-free survival rate, as compared with 5-FU alone in patients with curatively resected colorectal cancer (Mitomi *et al*, 1992). These results were promising evidence of survival benefits, and encouraged us to use oral PSK together with UFT as an adjuvant immunochemotherapy.

To investigate the benefits of immunochemotherapy with oral PSK plus UFT, we conducted a randomised, controlled trial comparing PSK and UFT in combination, with UFT alone, for 2 years, in cases of curatively resected stage II and III colorectal cancer. Here, we report the results of a 5-year follow-up.

## PATIENTS AND METHODS

This study was carried out in the Gunma Oncology Study Group (GOSG), which includes 19 affiliate hospitals of the Second Department of Surgery, Gunma University Hospital, Maebashi, Japan. Suitably qualified chief surgeons were trained in the Second Department of Surgery, Gunma University Hospital, in order to standardise surgical quality. Colon resection or tumour-specific mesorectal excision (MacFarlane *et al*, 1993) with D2 or D3 lymphadenectomy was performed. Once a patient was registered, his or her surgery and pathology reports, medical records, and laboratory tests, including haematology and blood chemistry, were reviewed by the quality-control committee of the GOSG. The ethics committee of each institution approved the protocol, and written informed consent was obtained directly from each patient before registration.

### Patients

All of the eligible patients had histologic confirmed colorectal cancer, were less than 75 years old, had measurable serum immunosuppressive acidic protein (IAP) levels, had a primary tumour at stage II or III, and underwent macroscopically curative resection. Patients were ineligible if they had undergone any radiotherapy, chemotherapy, or immunotherapy, or had multiple cancers, or severe complications. They were also ruled out if they showed any of the following abnormal findings: white blood cell count ⩽4000 mm^−3^, platelet count ⩽1 × 10^5^ mm^−3^; total protein level ⩽6.0 g dl^−1^; serum alanine or aspartate aminotransferase level ⩾100 IU l^−1^; or creatinine level ⩾1.5 mg dl^−1^. The subjects were randomly allocated to two groups immediately after surgery, using a centralised registration system. Assignment to the control or PSK-treatment group was made by referring to a pre-set list generated using a computer-assisted randomisation system.

Patients were allocated to PSK-treatment and control groups in the ratio of 2 : 1, because PSK plus chemotherapy (MMC plus 5-FU) had been shown to improve the disease-free and overall survival of patients with curative resection of colorectal or gastric cancers (Mitomi *et al*, 1992; Nakazato *et al*, 1994). The protocol called for a total of 240 patients to be accrued over 3 years in an allocation ratio of 2 : 1 of PSK-treated to control subjects (160 PSK and 80 control). This sample size would give an 80% probability of detecting a 15% difference in 5-year disease-free survival, with a two-sided *α* level of 0.05, which seemed both modest and reasonable with respect to the previous study (Mitomi *et al*, 1992).

### Study design

All registered patients received bolus injections of 12 and 8 mg m^−2^ MMC® (Kyowa Hakko, Inc., Japan) on postoperative days 1 and 2, respectively. The PSK group received oral PSK (3.0 g day^−1^) and UFT (300 mg day^−1^), starting 2 weeks after surgery and continuing for 2 years or until the diagnosis of tumour recurrence. The controls received UFT monotherapy.

The patients were followed up every 2 weeks in the first month after discharge, then once a month over the 2-year period of the study, and finally once every 2 or 3 months until 5 years after surgery. Patients were interviewed regarding drug intake on every visit, and randomly selected patients were interviewed by telephone. Drug diaries and pill counts were not used. Adverse effects were recorded according to WHO criteria, and, if these effects became intolerable, treatment was suspended until the effects subsided. Patients underwent ultrasonography, computer-aided tomography, and other procedures, as required, for every 6 months, to check for any evidence of cancer recurrence. When recurrence was noted, patients received an optimal treatment combination appropriate to their circumstances.

The disease-free and overall survival rates were the primary end points, and causes of death and recurrence were also assessed.

### Statistical analyses

Statistical analyses were carried out using the Statistical Analysis System software (SAS, version 8.2,. Cary, NC, USA). An intention-to-treat analysis was applied. Background factors were compared using the *χ*^2^ or Mann–Whitney *U*-test. Disease-free and overall survival curves were generated by the Kaplan–Meier method, and the log-rank test was used to compare the curves. Deaths without recurrence were censored. Proportional hazard regression analysis was applied to estimate the percentage reduction in risk of recurrence. Multivariate analyses using the Cox proportional-hazards model were applied to estimate the simultaneous effects of prognostic factors on survival. Variables were retained in the model if the associated two-tailed *P*-values were 0.05 or less.

## RESULTS

### Patient characteristics

A total of 207 patients were enrolled in the trial between October 1994 and March 1997. Slightly fewer patients were enrolled than the protocol sample called for, because the protocol study period was adhered to. Of these, 139 patients were allocated to the PSK group, and 68 to the control group. Two patients (1.4%) in the PSK group were ineligible: one had macroscopic noncurative tumours, and the other had multiple cancers. Consequently, 205 patients were analysed (137 PSK and 68 controls). All the patients were closely monitored throughout follow-up and no patients were lost during that time. No patient received the relevant treatment for more than 2 years. Table 1

**Table 1:** Patient baseline characteristics and macroscopic and histopathologic classification

		**Control group**		**PSK group**			
**Characteristic**	**Category**	**68**	**100%**	**137**	**100%**	***P*-value**	**Test**
Age (years)	25–49	8	11.8%	14	10.2%	0.433	*U*
	50–59	17	25.0%	33	24.1%		
	60–69	31	45.6%	57	41.6%		
	70–75	12	17.6%	33	24.1%		

Sex	Male	43	63.2%	71	51.8%	0.162	*χ*^2^
	Female	25	36.8%	66	48.2%		

IAP value	>500 *μ*g ml^−1^	21	30.9%	43	31.4%	1.000	*χ*^2^
	⩽500 *μ*g ml^−1^	47	69.1%	94	68.6%		

Location	Colon	34	50.0%	88	64.2%	0.071	*χ*^2^
	Rectum	34	50.0%	49	35.8%		

Tumour size (cm)	−3.9	16	23.5%	26	19.0%	0.356	*U*
	4.0–5.9	30	44.1%	59	43.1%		
	6.0–	22	32.4%	52	38.0%		

Residual tumour	R0	68	100.0%	136	99.3%	1.000	*χ*^2^
	R1	0	0.0%	1	0.7%		
	R2	0	0.0%	0	0.0%		

Pathologic primary tumour	T1	2	2.9%	4	2.9%	0.935	*U*
	T2	9	13.2%	8	5.8%		
	T3	49	72.1%	120	87.6%		
	T4	8	11.8%	5	3.6%		

Histolopathologic grade	G0	36	52.9%	45	32.9%	0.009	*χ*^2^
	G1	31	45.6%	81	59.1%		
	G2	1	1.5%	11	8.0%		

Pathologic regional lymph nodes	N0	40	58.8%	82	59.9%	0.555	*U*
	N1	12	17.6%	35	25.5%		
	N2	16	23.5%	20	14.6%		

Lymphatic invasion	L0	24	35.3%	39	28.5%	0.403	*χ*^2^
	L1	44	64.7%	98	71.5%		

Venous invasion	V0	34	50.0%	82	59.9%	0.190	*U*
	V1	33	48.5%	53	38.7%		
	V2	1	1.5%	2	1.5%		

Pathologic TNM stage*	I	8	11.8%	11	8.0%	0.914	*U*
	IIA	31	45.6%	68	49.6%		
	IIB	1	1.5%	3	2.2%		
	IIIA	3	4.4%	1	0.7%		
	IIIB	9	13.2%	34	24.8%		
	IIIC	16	23.5%	20	14.6%		

IAP=immunosuppressive acidic protein;

* TNM cancer staging according to AJCC (6th edition).

lists the baseline characteristics and histopathological classification of the subjects. There were no striking differences between the groups with respect to stratification factors or the histopathologic characteristics, including pathologic staging, pathologic regional lymph nodes, lymphatic invasion, venous invasion, and residual tumour excluding histopathologic grade. The histopathologic grade was higher in the PSK group than in the control group (*P*=0.009).

### Tumour recurrence and pattern of recurrence

**Figure 1:**
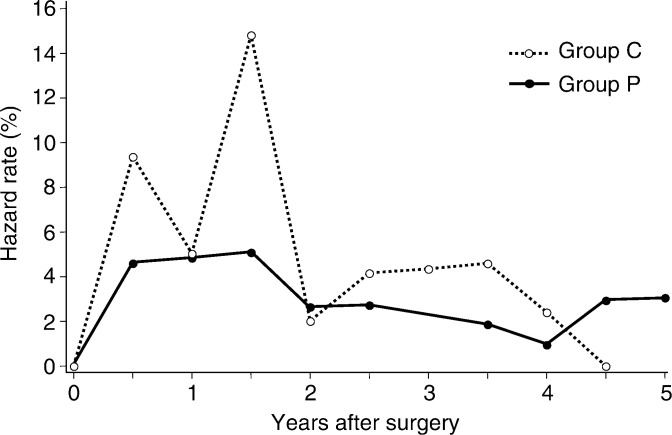
Recurrence hazard rate per 6-month interval.

In all, 57 patients had recurrences: 25 (36.5%; relative risk 0.66; 95% CI 0.45–0.98)) in the control group and 32 (23.3%; relative risk 1.26; 95% CI 0.98–1.63) in the PSK group (*P*=0.06; odds ratio 0.52; 95% CI 0.28–0.98). PROTEIN-BOUND POLYSACCHARIDE K administration reduced the absolute recurrence by 57.5%. The median time to recurrence was not significantly different (*P*=0.504) in the two groups: 2.0±1.4 years in the PSK group and 1.6±1.1 years in the control group. The 6-month rate of recurrence in the control group was higher in the first 2 years after surgery than later, whereas it was consistently low in the PSK group over the 5 years following surgery (Figure 1

).

Patients in the control group had 30 metastatic sites (relative risk 0.62; 95% CI 0.42–0.90), while patients in the PSK group had 37 sites (relative risk 1.31; 95% CI 1.03–1.67) (*P*=0.02; odds ratio 0.47; 95% CI 0.25–0.86). PROTEIN-BOUND POLYSACCHARIDE K prevented recurrence, particularly lung metastases (*P*=0.02; odds ratio 0.27; 95% CI 0.09–0.77), although there were no significant differences with regard to liver, peritoneal, local, and lymph node metastases (Table 2

**Table 2:** Tumour recurrence and pattern of recurrence

	**Control group**			**PSK group**					
	***N*=68**	**RR**	**95% CI**	***N*=137**	**RR**	**95% CI**	**OR**	**95% CI**	***P*-value**
No of patients with recurrence sites	25 (36.5%)	0.66	0.45–0.98	32 (23.3%)	1.26	0.98–1.63	0.52	0.28–0.98	0.06

Distant metastasis	21	0.67	0.45–0.99	26	1.27	0.96–1.67	0.52	0.27–1.02	0.08
Lung	10	0.49	0.32–0.76	6	1.85	0.97–3.50	0.27	0.09–0.77	0.02
Liver	9	1.04	0.58–1.85	19	0.98	0.75–1.29	1.06	0.45–2.48	1.00
Bone	1	0.33	0.27–0.40	0					0.72
Brain	1	0.33	0.27–0.40	0					0.72
Miscellaneous	0			1	0.67	0.61–0.73			1.00

Peritoneum	4	0.91	0.40–2.03	7	1.05	0.67–1.66	0.86	0.24–3.05	1.00
Lymph node	3	0.43	0.24–0.79	1	2.71	0.49–14.82	0.16	0.02–1.56	0.21
Local	2	0.83	0.28–2.46	3	1.12	0.54–2.30	0.74	0.12–4.53	1.00
Total	30	0.62	0.42–0.90	37	1.31	1.03–1.67	0.47	0.25–0.86	0.02

RR=relative risk, OR=odds ratio.

).

### Survival rates

**Figure 2:**
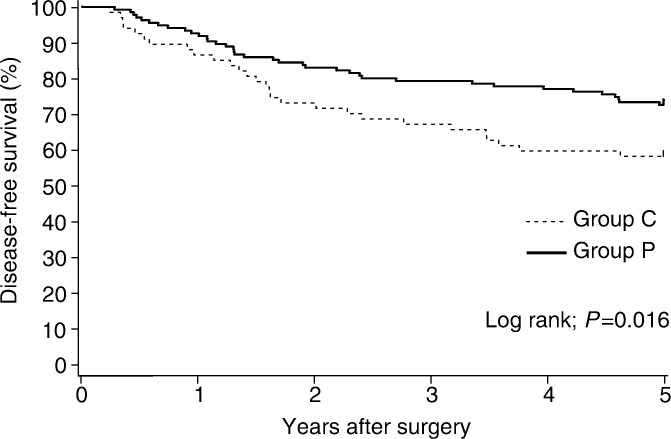
Disease-free survival (5-year) in all patients.

**Figure 3:**
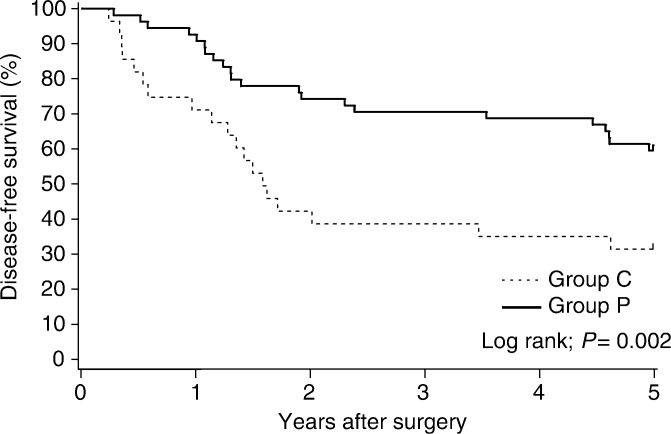
Disease-free survival (5-year) of patients with pathologic stage III cancer.

The mean disease-free survival time of the PSK *vs* control group was 50.3 months (95% confidence interval 47.2–53.4 months) *vs* 40.0 months (95% confidence interval 38.8–49.2 months) (*P*=0.031). The 5-year disease-free survival was 73.0% (95% CI 65.6–80.4%) with PSK and 58.8% (95% CI 47.1–70.5%) in the controls (*P*=0.016) (Figure 2

). The estimated overall reduction in the risk of recurrence with PSK therapy was 43.6% (95% CI 4.5–66.7%). In pathologic stage III patients, the 5-year disease-free survival benefit from PSK was significant: 60.0% (95% CI 47.1–72.9%) in the PSK group as compared with 32.1% (95% CI 14.8–49.4%) in the control group (*P*=0.002) (Figure 3

). The estimated overall reduction in the risk of recurrence with PSK therapy in pathologic stage III patients was 63.4% (95% CI 26.8–81.7%). No disease-free survival benefit was apparent in a subgroup analysis of colon and rectum, or pathologic stage II cancer.

All patients in whom there was recurrence received optimal chemotherapy, trans-arterial chemotherapy, and salvage surgery. In total, 21 salvage operations were carried out, for hepatic recurrence (10 patients), pulmonary metastasis (eight patients), local recurrence (two patients), or lymph nodes (one patient). A total of 21 patients (38.9%) remain alive: 12 (41.4%) patients in the PSK group and nine (36.0%) in the control. Three patients in the PSK group and two patients in the control group died from nonrelated disease.

**Figure 4:**
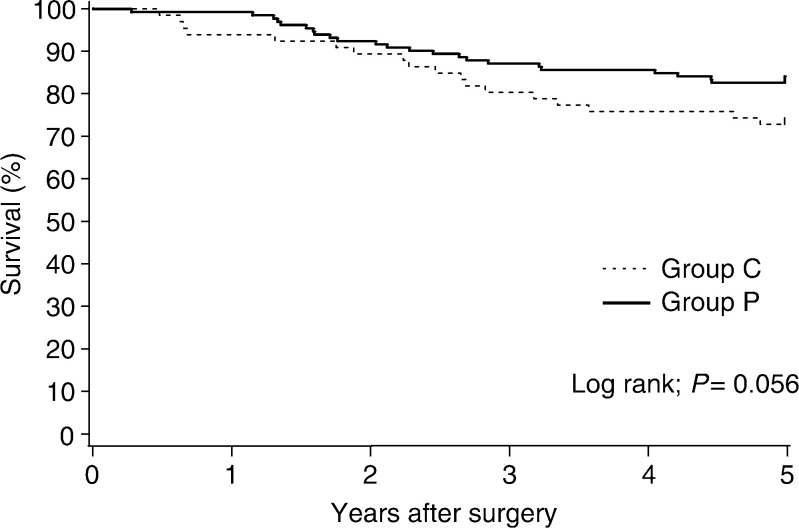
Overall survival (5-year) in all patients.

**Figure 5:**
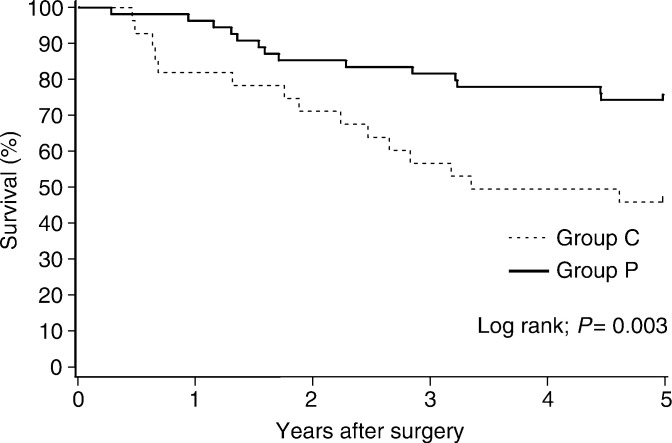
Overall survival (5-year) of patients with pathologic stage III cancer.

The mean survival time of the PSK group *vs* the control group was 54.0 months (95% confidence interval 51.6–56.4 months) *vs* 50.8 months (95% confidence interval 46.6–54.9 months) (*P*=0.150). The 5-year overall survival was 81.8% (95% CI 75.3–88.2%) in the PSK group and 72.1% (95% CI 61.4–82.7%) in the control group (*P*=0.056) (Figure 4

). The estimated overall reduction in mortality with PSK therapy was 40.2% (95% CI −12.5 to 68.3%). In pathologic stage III patients, the 5-year overall survival benefit from PSK was significant: 74.6% (95% CI 63.0–86.1%) in the PSK group, as compared with 46.4% (95% CI 28.0–64.9%) in the control group (*P*=0.003) (Figure 5

). The estimated overall reduction in mortality with PSK therapy in pathologic stage III patients was 66.2% (95% CI 24.8–84.8%). No selective survival benefit with PSK was apparent in a subgroup analysis of colon and rectum, or pathologic stage II cancer.

### Relative risks of recurrence according to proportional-hazards regression models

We analysed proportional-hazards regression models adjusted for sex, age, serum IAP levels, tumour locations, tumour size, pathologic primary tumour, presence or absence of regional metastases, and pathologic staging. It was found that lymphatic invasion, venous invasion, administration of PSK, pathologic primary tumour, and the presence or absence of regional metastases were each independently associated with an increased risk of recurrence. In the models, the presence of regional metastases (relative risk 2.973; 95% CI 1.712–5.165; *P*<0.001), omission of PSK (relative risk 2.109; 95% CI 1.221–3.633; *P*=0.007), and higher pathologic grade of primary tumour (relative risk 4.398; 95% CI 1.017–19.014; *P*=0.047) were each significant indicators for recurrence (Table 3

**Table 3:** Relative risks of recurrence according to proportional-hazards regression models

**Characteristic**	**Category**	**Harard ratio**	**95% CI**	***P*-value**
Sex	Male			
	Female	0.976	0.586–1.624	0.924

Age (years)	60–			
	–59	1.295	0.774–2.168	0.325

IAP value	>500 *μ*g ml^−1^			
	⩾500 *μ*g ml^−1^	1.357	0.777–2.370	0.284

Location	Colon			
	Rectum	1.446	0.866–2.483	0.154

Tumour size (cm)	−3.9			
	4.0–5.9	1.029	0.514–2.061	0.935
	6.0–	1.119	0.551–2.274	0.755

Primary tumour	T1, 2			
	T3, 4	4.398	1.017–19.014	0.047

Histopathologic grade	G0			
	G1, 2	1.380	0.764–2.496	0.286

Regional lymph nodes	N0			
	N1, 2, 3	2.973	1.712–5.165	<0.001

Lymphatic invasion	L0			
	L1	1.089	0.520–2.283	0.821

Venous invasion	V0			
	V1, 2	1.127	0.637–1.993	0.681

Group	PSK			
	Control	2.109	1.221–3.633	0.007

IAP=immunosuppressive acidic protein.

).

### Treatment compliance

In all, 89% of all patients were fully drug compliant: 87.6% in the PSK group and 91.2% in the control group. A total of 13 patients (6.3%) – nine (6.6%) in the PSK group and four (5.9%) in the control group – temporarily stopped taking the drugs because of adverse effects: gastrointestinal toxicity in five patients, myelosuppression in three patients, hepatotoxicity in two patients, dermatitis in two patients, and neurotoxicity in one patient. Another cause was bowel obstruction. Five patients refused treatment: four (2.9%) in the PSK group and one (1.5%) in the control group. Three patients received no treatment of MMC: two (1.5%) in the PSK group and one (1.5%) in the control group. Compliance with UFT and PSK was good, as confirmed by interviews and measurements of the urinary tegafur concentration carried out on 24 randomly selected patients between 11 and 24 months after surgery.

### Adverse effects

A total of 31 patients (15.1%) had grade 1 or 2 haematological or gastrointestinal toxicity. No grade 3 or 4 toxicity was observed. Gastrointestinal toxicity, such as loss of appetite, nausea, vomiting, diarrhoea, and stomatitis, was seen in 12 patients (5.8%). Other forms of toxicity included: haematological, 10 patients (4.9%); hepatotoxic, five patients (2.4%); neurotoxic, three patients, and dermatological, two patients. Toxicity occurred within 3 months after surgery, and was induced by the MMC and subsequent UFT treatments. MMC was definitively responsible for haematological toxicity in seven out of the 10 patients, because it occurred within 2 weeks of surgery.

### Second primary cancers

A total of 11 patients (5.4%) had second primary cancers: four (5.9%) in the control group and seven (5.1%) in the PSK group. Colorectal cancer occurred in five patients, while breast, lung, ovary, gall bladder, and pancreatic cancer occurred in one patient each. Curative-intent resections were performed on all patients, eight of whom have survived disease-free. One patient died from pancreatic cancer and another two died from the primary cancer.

## DISCUSSION

The study showed that oral PSK plus UFT reduced the risk of recurrence by 43.6% in patients with stage II or III, and reduced the overall death rate in patients with pathologic stage III colorectal cancer. In Cox proportional hazard models, the administration of PSK decreased the risk of recurrence and that of lymph node metastasis and a lower primary tumour.

After a 5-year follow up, the rate of recurrence was 36.5% with UFT monotherapy and 23.3% with UFT/PSK, with the majority of recurrences being distant metastasis. PROTEIN-BOUND POLYSACCHARIDE K achieved a 57.5% absolute reduction in recurrence at the 5-year follow-up. The recurrence rate was 34.9–37% for stages II and III, and 33.5–39.3% for stage III with 5-FU/LV and 5-FU/levamisole treatment (QUASAR, 2000; Porschen *et al*, 2001), whereas the rates were 49–60% without adjuvant chemotherapy. PROTEIN-BOUND POLYSACCHARIDE K reduced the risk of recurrence by 43.6% in stages II and III, and by 63.4% in pathologic stage III, which is equivalent to the reported 33.5–41% with 5-FU/levamisole or 5-FU/LV (Moertel *et al*, 1990; Francini *et al*, 1994; IMPACT, 1995).

In curative resected stage III colon cancer, the 4-year survival estimates range from 46 to 57% (Moertel *et al*, 1990; Francini *et al*, 1994; IMPACT, 1995). Adjuvant therapy with 5-FU/levamisole increased postoperative survival to 71% at 3 
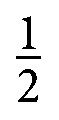
 years (Moertel *et al*, 1990). Further, adjuvant treatment with 5-FU modulated by LV increased 4-year survival to over 71–72% (Francini *et al*, 1994; IMPACT, 1995; QUASAR, 2000; Porschen *et al*, 2001). In our stage III patients, the 4-year disease-free and overall survival rates were 69.1 and 78.2% in the PSK group, as compared with 35.7 and 50% in the controls, respectively. The 5-year disease-free and overall survival rates were 60.0 and 74.6% in the PSK group, as compared with 32.1 and 46.4% in the controls, respectively. A randomised, double-blind study showed that the 5-year disease-free survival for PSK *vs* placebo was 38 *vs* 20%, and overall survival for PSK *vs* placebo was 50 *vs* 40%, respectively (Torisu *et al*, 1990). Survival with PSK/UFT treatment in pathologic stage III patients was comparable or superior to standard regimens of 5-FU/LV or 5-FU/levamisole (Moertel *et al*, 1990; Francini *et al*, 1994; IMPACT, 1995). However, survival with UFT monotherapy in pathologic stage III patients was worse, and was similar to that of untreated controls in standard regimen studies (Moertel *et al*, 1990; Francini *et al*, 1994; IMPACT, 1995). It is difficult to explain why survival in stage III was poor with UFT monotherapy; it might be explained by the difference in dose intensity of UFT. Tegafur/uracil was administered at a dosage of 600 mg body^−1^ in three divided doses daily (Malik *et al*, 1990) or 300 or 350 mg m^−2^ day^−1^ with LV (Saltz *et al*, 1995) in metastatic colorectal cancer. Our dose of UFT was less than that in those studies, despite the absence of LV modulation. There was a dose-dependent reduction in the odds of death for patients receiving 5-FU regimens (Zalcberg *et al*, 1996), while the dosage intensity was not a factor in the survival benefits (Higgins *et al*, 1976; Grage and Moss, 1981). Therefore, it remains speculative whether the poorer survival is explained by the difference in the dose intensity of UFT.

Studies may have shown a better tendency in overall survival, simply because the survival of pathologic stage II patients is so much better even without treatment. Another reason may be that the second-line treatments for recurrence, including chemotherapy and salvage surgical therapy, prolong the survival of these patients (Okumura *et al*, 1996; Goldberg *et al*, 1998; Saltz *et al*, 2000; Choti *et al*, 2002). All patients with recurrence received chemotherapy, trans-arterial chemotherapy, and salvage surgery (Tomizawa *et al*, in press). These treatments for recurrence certainly prolong overall survival and, in this era of active treatment, strategies for recurrence of disease-free survival may be more meaningful than overall survival, at least in the case of adjuvant chemotherapy for colorectal cancer.

The benefits of fluorouracil-based therapy for pathologic stage II colon cancer are unclear. While adjuvant active specific immunotherapy with BCG vaccine significantly lowered tumour recurrence in stage II colon cancer (Vermorken *et al*, 1999), we detected no beneficial effects of UFT/PSK with regard to the overall or disease-free survival rates in pathologic stage II cancer in this study, nor was it observed previously (Moertel *et al*, 1990; Mitomi *et al*, 1992). However, 20% of patients with pathologic stage II cancers die of recurrent disease, and lymph node micro-metastases are detected by molecular analysis in 54% of patients (Liefers *et al*, 1998). Molecular-based analyses would facilitate the selective use of adjuvant therapy and prevent unnecessary treatments.

Immunosuppressive acidic protein, isolated from the ascitic fluids of cancer patients, suppresses both phytohemagglutinin-induced lymphocyte blast formation and the mixed-lymphocyte reaction *in vitro* (Tamura *et al*, 1981). We expected the pre-operative IAP value to be a good predictor of PSK response, because a serum IAP value above 580 *μ*g ml^−1^, determined as an appropriate threshold value using Cox's proportional hazards model, has been reported to be a good indicator of enhanced survival in gastric cancer patients (Nakazato *et al*, 1994; Sakamoto *et al*, 1996). In Cox proportional-hazards regression models, the presence of regional metastases, omission of PSK, and higher primary tumour were each significant indicators of recurrence. Nevertheless, in this study, the pre-operative serum IAP level was not a significant predictor of survival.

The duration of adjuvant chemotherapy after colorectal cancer varies (Moertel *et al*, 1990; O'Connell *et al*, 1998; QUASAR, 2000; Porschen *et al*, 2001), and there is no consensus on the optimal duration of adjuvant chemotherapy. Currently, the standard adjuvant treatment for stage III colon cancer is 5-FU/LV for 6–8 months (Francini *et al*, 1994; IMPACT, 1995; O'Connell *et al*, 1998). When our study was designed, the optimal treatment duration was thought to be 2 years, because the risk of recurrence is greatest during that period and randomised controlled studies in which PSK and chemotherapy were administered for 18, 24, and over 36 months, showed a significant reduction in recurrence (Torisu *et al*, 1990; Mitomi *et al*, 1992; Nakazato *et al*, 1994, 1999). Indeed, in our study, 71% of cancers recurred during the first 2 years after surgery, and 85% recurred within 2 years and 6 months after surgery. The oral use of adjuvant chemotherapy with PSK and UFT has low toxicity, is simple, and does not require frequent treatment-related visits, thereby helping to ensure that patients receive treatment for 2 years.

Experimental studies have shown that PSK suppresses micro-metastases of the liver by enhancing the activities of NK cells and macrophages (Morinaga *et al*, 1994). It also reduces tumour cell invasiveness by downregulating several invasion-related factors, including TGF-*β*_1_, urokinase plasminogen activator (uPA), and matrix metalloproteinases (MMPs)-2 and -9, which are produced by tumour cells (Zhang *et al*, 2000). PROTEIN-BOUND POLYSACCHARIDE K restores host immune functions that are suppressed by tumour or antitumour chemotherapeutic agents, thereby producing IL-2 and *IFN γ*, and stimulating the activities of LAK and NK cells. PROTEIN-BOUND POLYSACCHARIDE K induces IL-1, IL-6, and IL-8 in peripheral mononuclear cells, as well as tumour necrosis factor and macrophage chemotactic factor at the tumour sites (Hirose *et al*, 1990; Ebina and Murata, 1992), thereby activating programmed cell death (Yefenof *et al*, 1995; Lacour *et al*, 2001). Therefore, the increased disease-free survival rates and marked reduction in lung metastases seen in this study may be evidence of immunological and biochemical modulation by PSK.

The toxicity of adjuvant chemotherapy regimens needs to be considered in selecting the optimal therapy. Oral PSK and UFT have less frequent and lower grade toxicity than 5-FU/levamisole and 5-FU/LV (Moertel *et al*, 1990; IMPACT, 1995; O'Connell *et al*, 1998; QUASAR, 2000; Porschen *et al*, 2001). Only 6.3% of the patients in this study discontinued treatment because of adverse drug effects. Of our patients, 15.4% showed grade 1 or 2 haematological and gastrointestinal toxic effects, while none had grade 3 or 4 effects. Drug compliance was better and was confirmed by interviews and measurements of the urinary tegafur concentration, as compared with well-tolerated standard regimens (Moertel *et al*, 1990; IMPACT, 1995; O'Connell *et al*, 1998; QUASAR, 2000; Porschen *et al*, 2001).

Finally, this study is limited in its application, because a small number of patients were enrolled, and the control arm did not consist of the standard adjuvant therapy with 5-FU/LV. Nevertheless, the results seem sufficiently promising to justify a larger scale study of adjuvant chemotherapy using oral PSK and UFT as an alternative to intravenous 5-FU/LV as the standard comparator arm. Future investigations will seek to progress beyond the era of 5-FU/LV.
